# Seroprevalence of Chikungunya Virus, Jamaica, and New Tools for Surveillance (Response) 

**DOI:** 10.3201/eid2808.221006

**Published:** 2022-08

**Authors:** Joshua J. Anzinger

**Affiliations:** The University of the West Indies, Kingston, Jamaica; The Global Virus Network Jamaica Affiliate, Kingston, Jamaica

**Keywords:** chikungunya, viruses, surveillance, Jamaica

**In Response:** I thank the authors for their favorable commentary (*1*) related to our recently published article (*2*). In their commentary the authors note that the low number of chikungunya cases captured through passive surveillance underrepresents the true burden of disease in Jamaica, particularly fatal infections during the 2014 chikungunya epidemic year (*3*).

Underreporting of chikungunya cases in Jamaica has been acknowledged and has multiple factors (*4*). Most chikungunya cases are not captured through a passive clinic-based surveillance (*5*), and in Jamaica most case-patients likely did not seek care at the advanced public health center passive surveillance sites. In addition, real-time PCR, the most sensitive diagnostic test type during acute infection, was highly limited in Jamaica during the 2014 chikungunya epidemic. For these reasons, identification of chikungunya cases through passive surveillance was expected to represent only a small fraction of the population.

It is possible that many excess deaths in Jamaica during 2014 were the result of chikungunya virus infections escaping surveillance. Chikungunya fatalities may be difficult to capture with limited surveillance capacity. Furthermore, chikungunya virus infections, particularly in the elderly, may exacerbate existing comorbidities and lead to extended hospitalization that could result in nosocomial infections; either event may prove fatal and ultimately be considered the cause of death (*6*).

During the COVID-19 pandemic in Jamaica, surveillance systems have been bolstered; the Ministry of Health and Wellness introduced broad community-based testing, many diagnostic laboratories have introduced real-time PCR testing, and the University of the West Indies has introduced next-generation techniques sequencing techniques for whole-genome sequencing of viruses. Further enhancing responses to emerging viruses, the University of the West Indies recently became a member of the Abbott Pandemic Defense Coalition that aims to increase virus surveillance and discovery (*7*). This increased infrastructure will likely improve surveillance for future viral epidemics in Jamaica.
